# Disseminated Intravascular Coagulation in Varying Age Groups Based on Clinical Conditions

**DOI:** 10.7759/cureus.24362

**Published:** 2022-04-21

**Authors:** Elizabeth Geyer-Roberts, Tanisha Akhand, Alexandra Blanco, Robin Jose, Nayeem Chowdhury, Michael Ea, Eric Gutierrez, Jacqueline Balbuena, Sophia Anagnostis, Claudia Henderson, Alexis Fazio, Alexander Burpee, Robin J Jacobs

**Affiliations:** 1 Medicine, Nova Southeastern University Dr. Kiran C. Patel College of Osteopathic Medicine, Fort Lauderdale, USA; 2 Integrative/Complementary Medicine, Nova Southeastern University Dr. Kiran C. Patel College of Osteopathic Medicine, Fort Lauderdale, USA; 3 Medical and Behavioral Research/Health Informatics/Medical Education, Nova Southeastern University Dr. Kiran C. Patel College of Osteopathic Medicine, Fort Lauderdale, USA

**Keywords:** circulatory, obstetrical dic, elderly persons, pregnancy, pediatrics & neonatology, neotates, disseminated intravascular coagulation (dic)

## Abstract

Disseminated intravascular coagulation (DIC) is a serious syndrome characterized by the systemic activation of blood coagulation resulting in the thrombosis of vessels leading to organ dysfunction and severe bleeding. When physicians try to treat DIC, it is imperative to diagnose and treat the underlying conditions. Anyone can be affected by DIC, but vulnerable groups such as pediatric populations, pregnant women and the elderly may be at higher risk. In this review, the current literature on DIC in pregnancy, the pediatric population, and the elderly is reported. This review also highlights the similarities and differences in the etiology, clinical presentation, diagnosis, and management of DIC in the aforementioned groups (i.e., pediatrics, pregnant women, and the elderly). Findings from this study may help increase awareness about various presentations of DIC in these groups to facilitate rapid recognition of symptoms leading to correct diagnoses.

## Introduction and background

Disseminated intravascular coagulation (DIC) is an occlusive process of the circulatory system that affects the microcirculation, generally occurring due to the effects of a concealed disease [1]. The root cause of the occlusive process is the activation of clotting mechanisms resulting in thrombus formation. Hemorrhage is another common clinical manifestation of DIC, which occurs due to the enhancement of fibrinolysis and the consumption of platelets and coagulation factors [2-4]. The combined effects of both thrombosis and hemorrhage result in the serious compromise of vital organs.

DIC is the simultaneous involvement of pro-thrombotic deposition in the microvasculature and increased action of fibrinolytic pathways [5,6]. Consequently, there is an increase in the consumption of coagulation factors, such as platelets, more rapidly than they can be produced, thereby resulting in thrombocytopenia. Increased fibrinolysis is evident by the increase in fibrin and fibrinogen split products, which results in an increased bleeding tendency. The most alarming repercussion of the concurrent thrombosis and increased bleeding is compromised organ function from the lack of oxygen supply and hemorrhage. The initiation of DIC is attributed to the result of systemic inflammatory activation secondary to an underlying disease process. The underlying disease process leads to the release of inflammatory mediators such as tumor necrosis factor (TNF)-⍺, interleukin (IL)-6, and IL-1 [7]. Additionally, intravascular webs, or “neutrophil extracellular traps,” have been attributed to the formation of thrombi in DIC and exacerbate the disease process [6].

This phenomenon can occur in both acute and chronic settings. Acute DIC has been seen as a complication of pregnancy, infection, tissue injury, shock, sepsis, and immunologic reactions. Chronic DIC can be evident in malignancy, hemangiomas, vascular and circulatory disorders, and liver disease. Additionally, DIC can affect all age groups, ranging from the pediatric population to the elderly population. In this review, we outline the current literature on DIC in pregnancy, the pediatric population, and the elderly population. This review also highlights the similarities and differences in the etiology, clinical presentation, diagnosis, and management of DIC in clinically vulnerable groups. It may also serve as guide to closing the current gap in knowledge and increasing awareness about the various presentations of DIC in vulnerable groups to facilitate rapid recognition of symptoms in order to lead to correct diagnoses.

## Review

Pediatric populations

In the pediatric population, the effects of DIC can be divided into two categories: neonates and toddlers. The two categories stem from the difference in underlying conditions that target these two subpopulations. In neonates, the most common causes of DIC are sepsis, perinatal birth asphyxia, respiratory distress syndrome, and meconium aspiration syndrome [8]. Pediatric DIC results in high mortality rates because of the delay of diagnosis, with neonates being the most susceptible [9,10].

Neonates

When physicians try to treat any level of pediatric DIC patients, it is important to target the underlying conditions [11,12]. Perhaps even more important than targeting the underlying conditions is an accurate initial diagnosis of DIC. The most crucial diagnostic tests to make a correct neonatal DIC diagnosis are the platelet count, D-dimer or FDP, PT, PTT, and fibrinogen [13]. The DIC diagnostic criteria of the Japan Society of Obstetrical, Gynecological & Neonatal Hematology, JSOGN, covers PLT, PT-INR, fibrinogen and d-dimer [14]. Neonates are given a score based on these factors ranging from 0 to 8. When analyzing these scores, they are usually grouped into those scoring ≥3 or <3. DIC scores of ≥3 were found to be associated with prenatal factors such as pregnancy-induced hypertension and placental abruption along with neonatal factors such as birth asphyxia, small for gestation age, low appearance, pulse, grimace, activity, and respiration (Apgar) scores, intraventricular hemorrhage, hemangioma, and hydrops [15].

In a study of 55 neonates that received treatment for suspected DIC from the Neonatal Intensive Care Unit of Fukushima Medical University Hospital, six died by day 28 [14]. The major deciding factor turned out to be the infants’ bleeding rates, as there were no significant differences in PT-INR, aPTT, FBG, D-dimer and AT. The study found that birth asphyxia was the leading underlying condition for infants diagnosed with DIC, with 53 out of the 55 neonates having birth asphyxia. There has been debate surrounding the treatment of infants diagnosed with DIC, specifically about differences in treatment based on the DIC scores received, and if the current JSOGN scoring guidelines are effective enough [14]. However, the one undebated topic is the deadliness of DIC in neonates.

In comparison to older children and adults, neonates, especially preterm neonates, are at greater risk for development of DIC due to limited reserves of coagulation factors including pro-coagulative and anti-coagulative factors [15]. In a study of 125 preterm infants, those born 24-27 weeks’ gestation were found to have the lowest levels of platelets and coagulation factors II, V, VII, and X. Amongst those infants, those who were small for gestational age and those with evidence of asphyxia with also found to have decreased platelets and coagulation factors. These data further emphasize the need to monitor for DIC amongst these populations at risk [15]. However, in the absence of risk factors and underlying pathologies, there is no evidence that the overall decreased hemostatic system in neonates alone prevents nor promotes thrombus formation. The neonatal hemostatic system remains of utmost importance, for in order to promptly and accurately diagnose neonatal DIC, coagulation laboratories ranges that specifically reflect neonatal levels are needed [15].

Toddlers and young children

In toddlers and young children, the most common causes of DIC are sepsis, trauma, and leukemia [12]. The most common systemic etiologies for sepsis were the respiratory system, followed by genitourinary, gastrointestinal, and central nervous system. The remaining three were associated with trauma, and all of them had subdural and/or intracranial bleeding that led to further serious complications [8]. In this patient population with leukemia, acute promyelocytic was the most prevalent subtype and there was a high incident of hemorrhage clinically.

Neisseria meningitidis induced Water-Friderichsen syndrome (WFS) is a fatal condition with a mortality rate of close to 50% within the 24 hours of being diagnosed. Water-Friderichsen syndrome is more common in children than adults, and it carries a risk of being associated with DIC. Pediatric patients usually present with vascular thrombosis characterized as necrosis of the skin plus bullae and vesicles. These clinical indications reflect the cumulative effects of hypercoagulation and hyperfibrinolysis [16]. After invasion into the bloodstream, the endotoxin of Neisseria meningitidis may trigger immune cell activation, leading to dysregulation of the immune system. ULvWF (ultra large von Willebrand factor) exocytosis leads to thrombocytopenia/endotheliopathy associated vascular microthrombotic disease that causes multiorgan failure. The endotoxin mediated pro-inflammatory cytokine activation leads to increased synthesis of cortisol, including adrenaline, by the adrenal gland. Considering adrenal insufficiency from WFS by checking ACTH in the patient is pertinent to administering sufficient parenteral hydrocortisone alongside saline with dextrose to restore intravascular volume, normalized serum sodium and blood glucose concentration [17]. Early clinical diagnosis and rapid administration of hydrocortisone can lower sepsis induced in DIC, specifically WFS.

In children and adults, trauma induced coagulopathy is primarily characterized by hyperfibrinolytic mechanisms that have limited plasminogen activator inhibitor (PAI) suppression. Acute phase of trauma contributes to massive bleeding and death. Blunt trauma causes a drastic increase in high mobility group box nuclear protein 1 (HMGB-1) and leukocyte-derived, platelet-derived microparticles. The anticoagulant pathways become dysregulated, and t-PA induced hyper fibrino(geno)lysis occurs. The unique property of fibrinogen cannot be replaced by infusion/transfusion and profuse bleeding continues [18].

A meta-analysis reported that acute promyelocytic leukemia (APL) was heavily reported under malignancy-induced DIC for children [19]. The hallmark clinical feature of this condition is hemorrhage that is due to hyperfibrinolysis. Marked increase in fibrin/fibrinogen degradation products (FDP)/D-dimer as well as hypofibrinogenemia explain the hyperfibrinolysis. Acute promyelocytic leukemia cells surface expression of annexin II aids 60-fold plasmin generation compared to non-APL cells. Moreover, the expressed annexin II are at higher levels that work more efficiently towards the conversion of plasmin from plasminogen. Mortality associated with APL occurs mostly during early chemotherapy interventions and has been steadily decreasing with the advent of new methods. In a report published by The Japanese Society of Hematology, all-trans retinoic acid (ATRA) introduction into anthracycline based chemo intervention reduced mortality in APL patients by downregulating annexin II [20]. Early Death during induction therapy still remains at around 30% and use of a human soluble recombinant thrombomodulin along with ATRA showed significant resolution and prevented exacerbations in DIC, specifically with WFS.

Pregnant women

Obstetrical DIC can become a complication of pregnancy, while differing mechanisms can lead to DIC during gestation. For example, postpartum hemorrhage (PPH) and placental abruption can lead to DIC in developed countries, while in developing countries hemolysis, elevated liver enzymes, and low platelets (HELLP) syndrome and preeclampsia are leading causes for DIC [21]. Other prevalent causes of obstetrical DIC include, but are not limited to, sepsis, septic abortion, retained stillbirth, acute fatty liver of pregnancy, amniotic fluid embolism, intrauterine infection, and pregnancy-induced hypertension [22].

Prompt detection and diagnosis of DIC in pregnancy becomes important to have favorable outcomes. Unfortunately, there is no singular clinical test to diagnose a patient with DIC, but different scoring systems, like the pregnancy modified DIC score created from components of The International Society on Thrombosis and Haemostasis (ISTH) DIC scoring system (a simple scoring system for the diagnosis of overt DIC that makes use of laboratory tests available in almost all hospital laboratories), are being developed [23]. The ongoing problem with scoring systems is that they are difficult to apply as pregnancy physiologically alters the clotting factors of the body [24].

In pregnancy, maternal leukocytes are at a higher rate of activation (expressing tissue factor [TF]). Typically, this is controlled during pregnancy through trophoblast properties. The trophoblast has two main functions: (1) to allow uninterrupted maternal blood flow and prevent clotting and (2) prevent bleeding at the placenta (maternal fetal interface). There is a need for uninterrupted blood flow but also a balance of not too much bleeding, thus syncytiotrophoblast (the premature placenta) will have endothelial cell like properties and express high TF. While on the contrary, the trophoblast (the cells outlining the newly formed zygote attempting implantation to the uterine wall) will synthesize protein C, protein S, protein Z, and placental protein 5 - all proteins designed to inhibit the coagulation cascade. The balance of proteins is overly sensitive and any disruption such as infection or amniotic fluid embolism could tip the scales and send the patient into DIC [23].

Lastly, the placenta trophoblast will increase its production of PAI-2 [23]. PAI-2 is a significant molecule that increases in the third trimester and at delivery, and participates in fetal and uterine tissue remodeling [24]. The increased concentration of secreted PAI-2, along with intracellular PAI-1, does not come with an associated increase in tissue plasminogen activator (tPA). In those situations, pregnant women become at higher risk for clotting; anything disrupting the integrity of the placental trophoblast is pivotal [23]. Any pathophysiology disrupting the trophoblasts can lead to a large release of TF, activating the coagulation cascade causing inflammatory responses which can eventually lead to DIC.

The pathophysiology of pregnancy clearly puts obstetric patients at risk and poses a huge complication for physicians since there is not a one-time special test to avoid this disease. However, a study from France was able to correlate the decrease in fibrinogen as a predictive factor in severe PPH (a common underlying cause of DIC) [25]. Of the 128 women in the study, 50 had severe PPH with corresponding fibrinogen levels at <2 g L-1. A simple assay like fibrinogen can be added to the management of high-risk obstetric patients to potentially decrease the chance of severe PPH and even DIC.

To date, there is scant published literature that explains maternal and neonatal outcomes, but poor maternal outcome is considered if DIC presents during delivery [26]. The most common causes of obstetrical DIC are PPH, placental abruption, and preeclampsia. It is during these traumatic and damaging events where the regulation of bleeding and clotting is lost [26]. One study reported that PPH resulted in 100% severe maternal morbidity, which included one or more of the following: emergency hysterectomy, acute tubular necrosis requiring dialysis, ICU admission, massive blood transfusion, and death [27]. Additionally, 49 of 151,678 deliveries were related to DIC yielding a case: fatality ratio of 1:16; there were three deaths due to severe coagulopathy from placental abruptions in a patient who refused blood products, severe coagulopathy from PPH, and an intracerebral hemorrhage following coagulopathy because of severe preeclampsia [27]. In all these cases of maternal death, the infants survived. However, the study reported 16 infant deaths (13 in utero and three as neonates). The highest rate of intrauterine fetal death was noted in mothers that had placental abruption. While the rates of DIC in the pregnant population may be low overall, the risk of mortality and morbidity associated with obstetrical DIC requires more investigation as much of the literature does not focus primarily on DIC in pregnancy, but instead focuses on the overview of DIC itself.

Elderly population

Common causes of DIC in the elderly population include sepsis and cancer, especially acute myeloid leukemia (AML) [28]. However, research on the outcomes of DIC for the geriatric age group with these conditions are currently limited. Although research is scarce, DIC in elderly individuals can arise as a complication of a number of different conditions.

Heat illness, which elderly individuals are at increased risk, is one example that can be complicated by DIC [29]. One study done in Japan found that older individuals suffering from heat illness had particularly worse outcomes when accompanied with DIC. Underlying conditions and the number of impaired organs the patients presented with could have played a role. However, DIC was found to be a major complication in these patients [30]. Further research in the early management of patients with possible DIC can prove beneficial and can possibly improve outcomes in the patient population.

COVID-19 has become a major public health emergency, with the elderly population being particularly susceptible. With severe fulminant cases of COVID-19, activation and consumption of clotting factors can lead to DIC [28]. This is associated with the marked upregulation of plasma cytokines as seen in a cytokine storm. In addition, normal aging is linked with a low-grade, systemic, pro-inflammatory state, a phenomenon known as “inflammaging.” This is a significant risk factor for morbidity and mortality in the elderly, as a systemic inflammatory state and coagulation disorders such as DIC are interrelated. Inflammaging relates to an increase in pro-inflammatory cytokines, particularly IL-6, TNF-α, and IL-1β [28]. Furthermore, PAI-1 expression is increased in elderly individuals which inhibits fibrinolysis and thus adds to the risk of a thrombotic event.

Elderly male patients with COVID-19 are more likely to develop cytokine storms than females, which makes them more susceptible to coagulation abnormalities. Additionally, laboratory testing in elderly males show that they have longer prothrombin time (PT) and increased fibrinogen compared to females. It is important to note that while COVID-19 coagulopathy shares many features with DIC, it also has certain laboratory features that are different than the “classical” presentation of DIC as seen in sepsis-associated DIC [31]. Similarities between COVID-19 coagulopathy and classical DIC include an abnormally elevated D-dimer level, thrombocytopenia, and prolonged coagulation times (such as PT). However, key differences between COVID-19 coagulopathy and the more typical sepsis-associated DIC include more severe thrombocytopenia and much lower levels of coagulation inhibitors such as antithrombin and protein C in sepsis-associated DIC.

## Conclusions

DIC is a deadly condition that targets specific vulnerable populations. This review explored the effects and outcomes of DIC within pediatric, pregnant, and elderly populations. When addressing DIC in the pediatric population, the rapid growth of the patients makes it necessary to divide the population into two subpopulations: neonates and toddlers/children. Neonates are the most susceptible out of the pediatric population, with birth asphyxia being the number one cause. The determining factor of survival was the bleeding rate, and those neonates with lesser rates of bleeding survived longer than those with greater rates of bleeding. In toddlers/children, the number one cause was sepsis. In pregnant women, the poor maternal outcome was observed when DIC was presented at delivery. The most common causes of obstetrical DIC are PPH, placental abruption, and preeclampsia. Elderly populations are particularly at risk for DIC due to the low-grade, systemic, pro-inflammatory state associated with aging. DIC in the elderly is most often caused by sepsis but has most recently been tied to COVID-19, as COVID-19 also presents with coagulopathy. A risk factor for the elderly is being male, as males are more likely to develop cytokine storms than females, which makes them more susceptible to coagulation abnormalities. In all these populations, a crucial factor is a quick and correct diagnosis of DIC. In order to decrease morbidity and mortality, the possibility of DIC must always be considered when treating vulnerable populations as mentioned above.
